# A Fleshy Mystery: Prurigo Nodularis

**DOI:** 10.7759/cureus.65125

**Published:** 2024-07-22

**Authors:** Jianna Bona, Micah Pippin, Logan Atkins, Majeed Al-Quraishi

**Affiliations:** 1 Family Medicine, Louisiana State University (LSU) Health Shreveport/Rapides Regional Medical Center, Alexandria, USA; 2 Pathology, Louisiana State University (LSU) Health Shreveport/Rapides Regional Medical Center, Alexandria, USA

**Keywords:** irritable bowel syndrome, somatic disorders, atopic disease, hiv testing, prurigo nodularis (pn)

## Abstract

Prurigo nodularis is a chronic dermatologic condition typically presenting as firm, dome-shaped pruritic nodules of various sizes that are often symmetrically distributed on extensor surfaces of the extremities. The diagnosis is clinical and based on a history of chronic, severe pruritis in the setting of characteristic, excoriated lesions. Treatment is difficult and aimed at reducing skin irritation and pruritus. Quality of life is impacted due to the chronic, intractable, and relapsing nature of the disease. Here, we report a case of prurigo nodularis diagnosed in the outpatient clinic setting. This paper aims to report the patient's clinical history, presentation, and histopathologic findings, as well as present a literature review to determine the significance of this case and the approach to ongoing management.

## Introduction

Prurigo nodularis is an uncommon, chronic skin condition that usually manifests as firm, dome-shaped pruritic nodules of various sizes that are often symmetrically distributed on extensor surfaces of the extremities [1]. The diagnosis is clinically based on the history of chronic, severe pruritis in the context of characteristic excoriated lesions. However, a skin biopsy may be helpful to reveal orthokeratosis, epidermal hyperplasia, diminished nerve fiber density, and inflammatory dermal infiltrates [2]. Management primarily includes treatments to reduce skin irritation and pruritus [3]. While many therapeutic options are available, the relapsing nature of the disorder results in chronic, recurring eruptions and pruritus. A multidisciplinary approach may be beneficial to not only address the integumentary manifestations of the disorder but also identify and manage extra-dermatologic sequelae, such as diminished mental health. Here, we report a case of prurigo nodularis refractory to supportive care with plans to initiate a biologic agent.

## Case presentation

**Figure 1:**
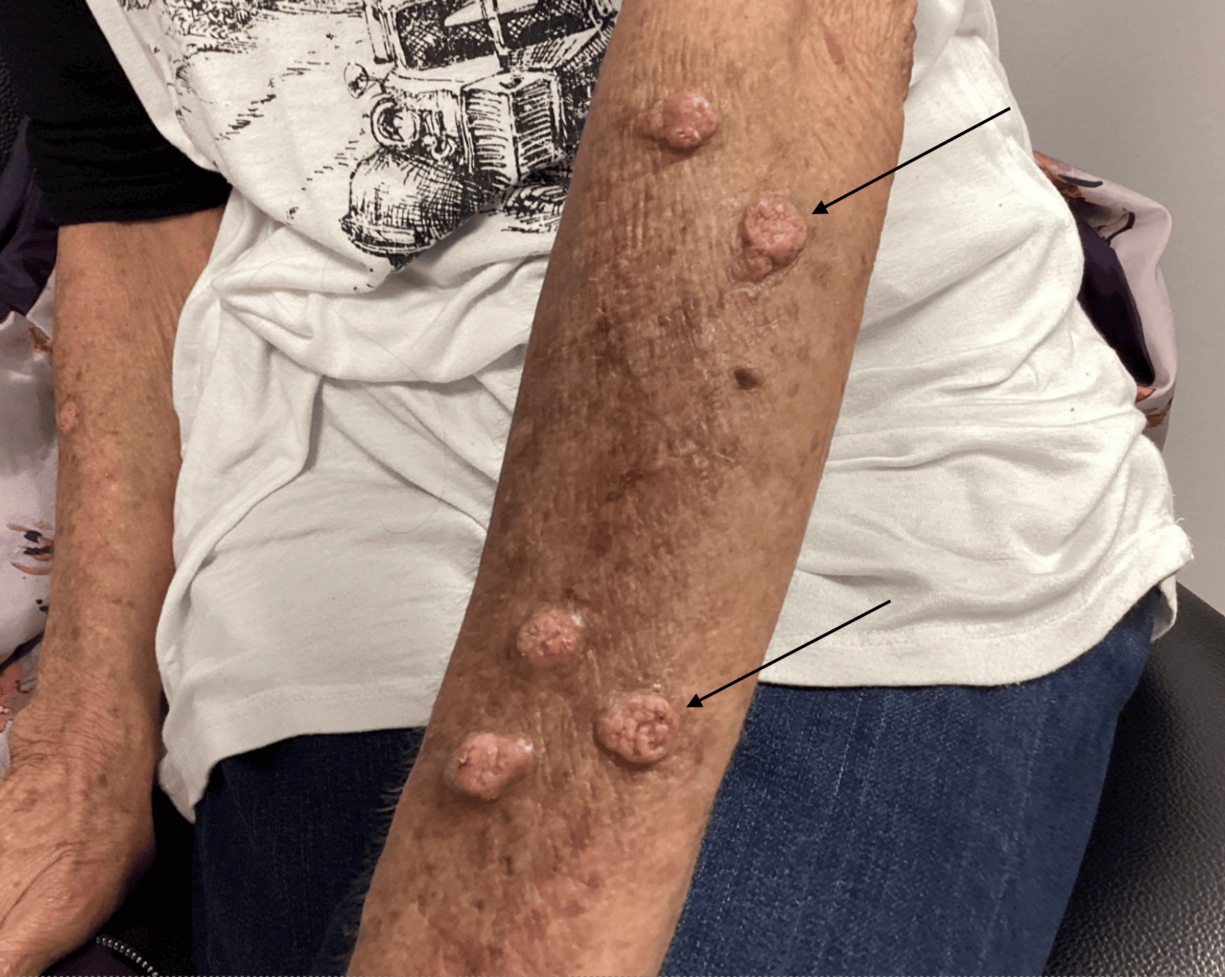
Nodular eruption of left upper extremity

The patient was a 64-year-old female with a past medical history significant for hypertension, gastroesophageal reflux disease, anxiety, chronic obstructive pulmonary disease, and previously excised facial skin cancer who presented to the outpatient clinic for evaluation of various skin lesions (Figure 1).

**Figure 2:**
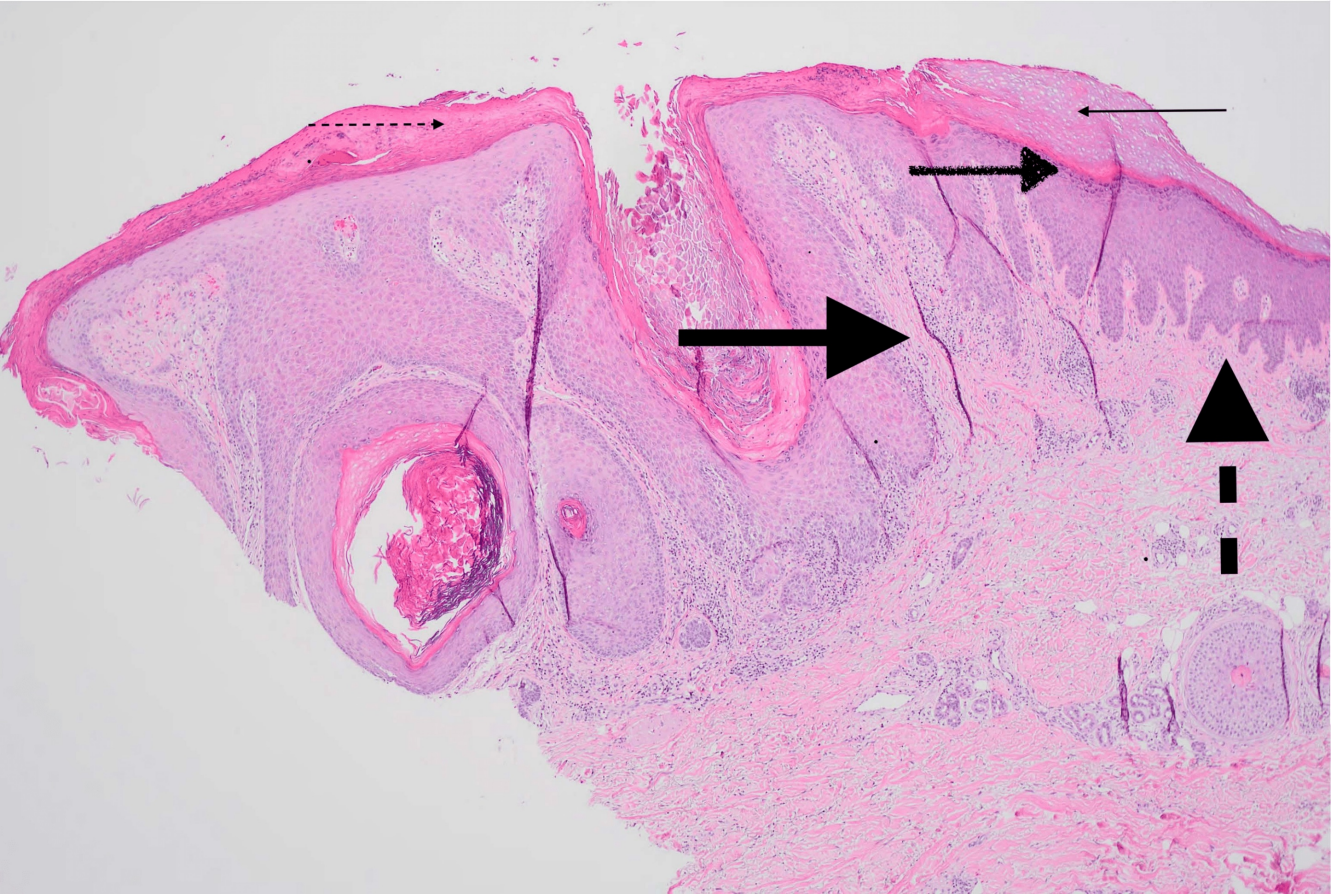
Skin biopsy demonstrating typical features of prurigo nodularis Small solid arrow - orthokeratosis; Small dashed arrow - hyperkeratosis; Medium brushed arrow - hypergranulosis; Large solid arrow - verticle streaking of collagen bundles; Large dashed arrow - irregular acanthosis

She stated the lesions had been present for several months and usually erupted in places where she had "sun spots." The patient endorsed a history of exposure to multiple chemicals, including Roundup and Kennel Dip, at the time the lesions began. Associated symptoms included itching, and the patient often scratched at the nodules. She was previously treated with triamcinolone ointment without improvement. Daily products used included Gain or Tide pods, Olay body wash, Hibiclens, and Cerave moisturizer. She smoked a few cigarettes per day and denied illicit or recreational drug use. She denied recent travel, illness, or close contact with individuals with similar lesions. Of note, the patient reported a history of Crohn's disease, which was not being treated with pharmacotherapy. Colonoscopic evaluation suggested inflammation similar to Crohn's disease; however, the gastroenterologist did not see any evidence of active disease. On physical examination, the patient was thin and resting comfortably. She had numerous cracked and waxy-appearing raised nodules varying from 5-15 mm overlying all extremities without surrounding erythema or drainage. Skin appeared overall aged with many hyperpigmented macules, various seborrheic keratoses, and scarring throughout. Laboratory findings revealed a leukocyte count of 14.0 WBCs per microliter (normal 4.5-11 WBCs per microliter) with 71.5% granulocytes, an estimated glomerular filtration rate (GFR) of 76 mL/min, human immunodeficiency virus (HIV) one and two antigen and antibody combination negative, hepatitis B surface antigen negative, hepatitis C antibody negative, rapid plasma reagin (RPR) non-reactive, antinuclear antibody (ANA) positive, anti-DNA antibodies negative. A 6 mm punch biopsy was performed to evaluate the uncertain nature of the skin lesions and concern, given the patient's previous history of skin cancer. The pathology report revealed hyperkeratosis, orthokeratosis, hypergranulosis, irregular acanthosis, and vertical streaking of collagen bundles (Figure 2). No atypia was present.

The differential diagnosis included prurigo nodularis, acquired reactive perforating dermatoses, pemphigoid nodularis, nodular scabies, hypertrophic lichen planus, and multiple keratoacanthomas.

The topical steroid triamcinolone, 0.1% cream, was initiated, with instructions to apply it to the affected areas twice daily. The patient was also prescribed the antihistamine hydroxyzine to aid with continuing pruritus. On one-month follow-up, the patient's lesions had decreased in size, and the itching was improved. After a second month of continued treatment, the patient reported returning symptoms and dissatisfaction with the current management plan. Dupilumab (Dupixent), a monoclonal antibody that blocks interleukin-4 and interleukin-13, was begun at 300 mg subcutaneously every other week. Three months of this regimen did not provide adequate relief. Methotrexate 12.5 mg orally was then initiated for recalcitrant disease. Folic acid was co-administered with methotrexate, and the patient's metabolic panel and complete blood count were monitored at monthly follow-ups. The patient tolerated this immunosuppressive therapy well, improving her lesions and pruritis. Her follow-up appointments were moved to every three months, and she enjoyed sustained improvement in symptoms and quality of life. 

## Discussion

Prurigo nodularis typically presents as firm, dome-shaped pruritic nodules ranging in size from millimeters to several centimeters. Lesions are often symmetrically distributed on extensor surfaces of the extremities. Nodules may be flesh-colored, erythematous, or hyperpigmented. Lesions range in number from a few scattered nodules to hundreds of nodules. Difficult-to-reach areas, including the mid-back, are often spared [1,2]. Prurigo nodularis is a clinical diagnosis based on a history of long-standing severe pruritus in the setting of characteristic excoriated nodular lesions that are symmetrically distributed. A skin biopsy may be indicated in cases where the clinical diagnosis is uncertain or the patient responds poorly to first-line treatment options. Initial laboratory and imaging evaluation may include a complete blood count (CBC), liver function tests (LFTs), blood urea nitrogen (BUN), creatinine, thyroid stimulating hormone (TSH), HIV, urinalysis, chest radiography, and stool ova and parasite examination. Histopathologic features generally include thick compact orthohyperkeratosis, irregular epidermal or pseudoepitheliomatous hyperplasia, focal parakeratosis with irregular acanthosis, diminished nerve fiber density, and nonspecific dermal infiltrate containing lymphocytes, macrophages, eosinophils, and neutrophils. Hair follicles may be seen in the center of the lesion [2]. The exact pathogenesis of prurigo nodularis remains unclear. However, a few mechanisms have been hypothesized, including increased nerve fibers in the papillary dermis, cutaneous small fiber neuropathy, and type 2 helper T (Th2) cytokines [4-6].

The exact incidence and prevalence statistics are unknown; however, the estimated prevalence is 72 per 100,000 people [2]. Prurigo nodularis primarily affects older adults, with a median age of 62 [2]. In the United States, prurigo nodularis more commonly affects African Americans [7]. Prurigo nodularis occurs in approximately five percent of patients with HIV infection [7].

Coexisting pruritic skin conditions such as eczema or xerosis may be the initial cause of itching, and approximately half of the affected patients have a history of atopic dermatitis. Diabetes, chronic kidney disease, cardiovascular disease, hepatitis C, celiac disease, psychiatric disorders, sleep disorders, and emotional stress have been reported with high frequency in patients with prurigo nodularis. Conditions that are associated with alterations in CNS pain processing, including fibromyalgia, interstitial cystitis, and irritable bowel syndrome, have also been linked with a higher incidence than the general population. Rarely, prurigo nodularis may be the presenting symptom of a systemic disease such as HIV (CD4+ count less than 200), mycobacteria, a parasitic infection, or lymphoma [8,9].

Treatment of prurigo nodularis is challenging and requires a multifaceted approach. The standard of care includes general measures to reduce skin irritation and pruritus and topical or systemic therapies to interrupt the itch-scratch cycle and flatten the skin lesions. Gentle skin care with mild cleansers and emollients can be used multiple times daily to soothe and moisten skin. Treatment of any underlying compulsive behavior, such as skin picking, should be addressed. First-generation antihistamines taken at bedtime may be helpful in controlling nocturnal pruritus. Selective serotonin reuptake inhibitors (SSRIs) and tricyclic antidepressants (TCAs) may also be used for chronic pruritus [3,9]. Treatment of limited disease may include super-potent (group one) topical corticosteroids applied nightly under occlusive plastic wrap for at least two to four weeks or twice daily if an occlusive wrap is not utilized. Intralesional triamcinolone injections every four weeks until pruritis subsides and nodules flatten have also been effective for some patients. Other topical therapies such as capsaicin, calcineurin inhibitors, and vitamin D analogs have been used with some success in patients with limited disease [3,10]. Treatment of widespread or refractory disease may include phototherapy and biologic agents. One possible option is narrowband ultraviolet B phototherapy administered two to three times per week for up to 10 weeks, combined with topical corticosteroids [11]. Dupilumab (interleukin-4 receptor inhibitor) has been shown to be effective, especially in those with underlying eczema. Dupixent is initially administered subcutaneously at 600 mg, followed by 300 mg every two weeks. Other immunosuppressants, including low-dose methotrexate (7.5-20 mg weekly) or oral cyclosporin (3-5 mg/kg daily), may be beneficial [12]. Prurigo nodularis is a chronic and often intractable disease with a high risk of recurrence. Life expectancy is usually not affected; however, the condition often lasts for years, profoundly impacting quality of life. Complete resolution of lesions is rare, even after successfully interrupting the itch-scratch cycle.

## Conclusions

Prurigo nodularis typically presents as firm, dome-shaped pruritic nodules of various sizes that are often symmetrically distributed on extensor surfaces of the extremities. The diagnosis is clinical and based on the history of chronic, severe pruritis in the setting of characteristic, excoriated lesions. A skin biopsy may be necessary for the diagnosis, revealing orthokeratosis, epidermal hyperplasia, diminished nerve fiber density, and inflammatory dermal infiltrates. Prurigo nodularis more commonly affects patients with coexisting eczema and atopic dermatitis. Systemic diseases, including HIV and malignancy, should be ruled out with basic laboratory tests and imaging. Treatment is difficult and aimed to reduce skin irritation and pruritus. Topical and systemic therapies may be used to interrupt the itch-scratch cycle and flatten skin lesions. Biologic agents such as Dupixent and phototherapy, in combination with high-potency topical steroids, are the mainstays of treatment for widespread disease. Quality of life is often impacted due to the chronic, intractable, and relapsing nature of the disease process.
